# Free fatty acids profile among lean, overweight and obese non-alcoholic fatty liver disease patients: a case – control study

**DOI:** 10.1186/s12944-017-0551-1

**Published:** 2017-09-04

**Authors:** Rennan Feng, Chao Luo, Chunlong Li, Shanshan Du, Akinkunmi Paul Okekunle, Yanchuan Li, Yang Chen, Tianqi Zi, Yucun Niu

**Affiliations:** 1 Department of Nutrition and Food Hygiene, School of Public Health, Harbin Medical University, 157 Baojian Street, Nangang District, Harbin, Heilongjiang Province 150081 China; 2 STD & AIDS Center, Harbin Center for Disease Control and Prevention, Harbin, Heilongjiang Province 150056 China; 3 Department of General Surgery, the Second Affiliated Hospital of Harbin Medical University, Harbin, Heilongjiang Province 150081 China

**Keywords:** FFA, Body weight, NAFLD, GC-MS, Myristic acid, Palmitoleic acid

## Abstract

**Background:**

Non-alcoholic fatty liver disease (NAFLD) given its association with obesity and diabetes may perhaps exert distinct free fatty acids (FFA) pattern, but the understanding of this phenomenon is limited. To this effect, we evaluated FFA profiles among healthy subjects and NAFLD patients stratified by body weight, to identify FFA valuable for early diagnosis of NAFLD.

**Methods:**

Serum FFA profiles of healthy and NAFLD (lean, overweight and obese) subjects was determined using gas chromatography–mass spectrometry (GC–MS) and distinctions in FFA patterns were evaluated using one-way ANOVA while Receiver operating characteristics (ROC) and logistic regression models were used to explore FFA significant for diagnosing NAFLD.

**Results:**

NAFLD patients presented significantly higher (*P* < 0.05) serum FFA profiles compared to healthy controls (HC). While total FFA profiles were insignificantly different between lean (2093.33 ± 558.11 μg/ml) and overweight (2420.81 ± 555.18 μg/ml) NAFLD patients, obese NAFLD (2739.01 ± 810.35 μg/ml) presented most significantly elevated (*P* < 0.05) total FFA profiles compared with HC. Of the four FFA; myristic acid (14:0), palmitoleic acid (16:1), γ-linolenic acid (γ-18:3) and cis-7,10,13,16,19-docosapentaenoic acid (22:5), selected in ROC analysis given their high Youden’s index and AUC, only 14:0; 5.58(1.37, 22.76) and 16:1; 4.36(1.34, 14.13) had statistical significant odd ratios.

**Conclusion:**

Our findings suggest 14:0 and 16:1 are promising for early diagnosis of NAFLD.

**Electronic supplementary material:**

The online version of this article (doi:10.1186/s12944-017-0551-1) contains supplementary material, which is available to authorized users.

## Background/introduction

Serum free fatty acids (FFA) perhaps reflect the imbalance between release and uptake of triglyceride (TG) in muscle, heart, liver, and other tissues [1], and its high level often result in TG synthesis alongside elevated concentration of very low-density lipoprotein cholesterol [2]. On the other hand, insulin probably decreases FFA by inhibiting hydrolysis and promoting storage of TG [3]. Increased serum FFA levels are often accompanied by insulin resistance (IR), leading to the onset of metabolic disorders, such as obesity, dyslipidemia, type 2 diabetes (T2DM) and cardiovascular diseases [4, 5].

Elevated plasma FFA in NAFLD patients has been mostly clarified to be closely associated with metabolic changes in the liver [6–8]. For example, sodium palmitate increases intracellular lipid accumulation and induce lipoapoptosis in human liver [9, 10]. Also, long-chain *n*-3 polyunsaturated fatty acids (LCPUFA) are beneficial against fat deposition and liver steatosis. Depletion of LCPUFA (n-6 and n-3 series) has been linked with lipid accumulation [11, 12]. Barr et al. [13] evaluated the metabolic distinctions between NAFLD and normal liver using animal models and found significant differences between their FFA profiles. Though saturated FFA has been reported to be a risk factor for NAFLD [14], it is yet to clearly established whether NAFLD patients (especially by their body weights) express significant distinct patterns of FFA profiles from healthy subjects.

Even though obesity is a well-known risk factor for the onset of NAFLD, yet NAFLD could also be observed in normal weight individuals (commonly called lean-NAFLD) with unique metabolic characteristics such as lower fasting blood glucose, HbA1c, blood pressure and better insulin sensitivity when compared with obese patients. It has also been considered as a frequent cause of cryptogenic liver disease in normal weight subjects [15]. To date, no study has explored the potential distinctions in FFA patterns of NAFLD patients especially by body weights as well as the diagnostic value of FFA in lean-NAFLD. Exploring simple, timely and cost effective way for disease diagnosis at an earlier stage is of great significance in managing morbidity and improving health-related quality of life.

Against this background, we applied metabonomics to measure the FFA profile of NAFLD patients primarily to evaluate distinctions in FFA profiles of NAFLD patients by body weight as well as promising FFA for timely diagnosis of NAFLD.

## Methods

### Study population

Of the 80 healthy individuals and 240 NAFLD patients recruited at Physical Examinations Center of the Second Affiliated Hospital, Harbin Medical University between March 12, 2013 and April 20, 2013, individuals with a history of alcohol drinking, malignancies, pregnancy, and long-term use of estrogens, tamoxifen or corticosteroids were excluded from the current study [Additional file 1: Figure S1]. In addition, individuals diagnosed with other diseases/infections aside NAFLD only were excluded from our study.

#### Selection criteria

Healthy controls (HC): Body mass index (BMI); 18.5–24.0 kg/m^2^, with normal liver biopsy and without a clinical diagnosis of any known liver disease or another disease(s).

Lean NAFLD: Fulfils the diagnostic criteria for NAFLD only, without any history or diagnosis of any other disease(s) and BMI: 18.5–24.0 kg/m^2^.

Overweight NAFLD: Fulfils the diagnostic criteria for NAFLD only, without any history or diagnosis of any other disease(s) and BMI: 24.0–28.0 kg/m^2^.

Obese NAFLD: Fulfils the diagnostic criteria for NAFLD only, without any history or diagnosis of any other disease(s) and BMI ≥ 28.0 kg/m^2^.

In all, 66 HC and 170 NAFLD patients; lean – NAFLD (67), overweight – NAFLD (55) and obese – NAFLD (48) met the inclusion criteria and were included in final analysis. Also, the Ethics committee of Harbin Medical University approved the study (2013019) and written informed consents were obtained from all respondents prior to their voluntary participation in the study.

### Demographic, anthropometric and biochemical measurements

Subjects who participated in the physical examination were interviewed by trained interviewers to complete a questionnaire that included questions such as; age, sex, history of disease(s), drug use, regular exercise, and alcohol consumption. Regular exercise was categorised as ‘vigorous’ or ‘moderate’ or ‘none’ where respondent reportedly engaged in any form of strong/hard physical exercise/labour (to promote substantial increase in energy expenditure or maintain one or more components of physical fitness) at leisure or occupational time for ≥3 times or once or none per week respectively. Habitual alcohol intake was assessed using the amount of alcohol intake of respondents multiplied by the frequency of consumption.

Weight, height, waist circumference (WC), body fat and blood pressure (BP); including systolic BP (SBP) and diastolic BP (DBP) were measured by well-trained examiners as reported in [16]. Body mass index (BMI) was calculated dividing body weight (kg) by the square of height (m) and cut-off points according to [17]. All respondents donated blood samples after ≥10 h overnight fasting for biochemical measurement; including fasting blood glucose (FBG), fasting insulin (F-insulin), total cholesterol (TC), high-density lipoprotein cholesterol (HDL-C), low-density lipoprotein-cholesterol (LDL-C), triglycerides (TG), aspartate aminotransferase (AST), alanine aminotransferase (ALT), γ-glutamyl transferase (GGT), alkaline phosphatase (ALP), serum creatinine (Crea), blood urea nitrogen (BUN) and serum uric acid (UA), while HOMA-IR was estimated as previously described in [18]. Additionally, blood samples were centrifuged at 2500×g for 15 min at room temperature within 30 min of collection, and the supernatant was kept at −80 °C until FFA measurements.

### FFA measurement

Serum FFA profile was measured using gas chromatography–mass spectrometry method (GC-MS) described previously in [19].

Firstly, we purchased 12 fatty acid standards from Sigma (St Louis, MO, USA, ≥99% purity), including myristic acid (14:0), palmitic acid (16:0), palmitoleic acid (16:1n-7), stearic acid (18:0), oleic acid (18:1n-9), linoleic acid (18:2n-6), linolenic acid (18:3n-3), γ-linolenic acid (γ-18:3n-6), arachidonic acid (20:4n-6), cis-5,8,11,14,17-eicosapentaenoic acid (20:5n-3), cis-7,10,13,16,19-docosapentaenoic acid (22:5n-6), and cis-4,7,10,13,16,19-Docos-ahexaenoic acid (22:6n-3). Stock solutions of all the above fatty acids, internal standards (heptadecanoic acid) and calibration samples (spiking with 12 different concentrations of fatty acids standards) were prepared.

Briefly, aliquots (200 μL) of serum were spiked with an internal standard working solution (200 μL heptadecanoic acid C17:0200 μg/mL), and 1 mL 0.05% H_2_SO_4_ was added to deposit protein. FFA were extracted using 3 mL ethyl acetate, mixed for 60s (using a vortex mixer) and centrifuged at 4000×g for 10 min at room temperature. The ethyl acetate phase was evaporated to dryness under N_2_. Following the addition of 2 mL 10% H_2_SO_4_-CH_3_OH and incubation in 62 °C water bath for 2 h, 2 mL saturated sodium chloride and 2 mL hexane was sequentially added and mixed for 60s to obtain the fatty acid methyl esters. Samples were evaporated to dryness under N_2_ gas, and 100 μL hexane was added to each tube prior to analysis.

FFA concentrations of all standard and serum samples were determined using TRACE gas chromatograph with a Polaris Q mass spectrometer (Thermo Finnigan, Austin, TX, USA) (GC–MS). A split injector (the split ratio being 1:10) at 230 °C was used to add the sample (1.0 μL) onto a J&W DB-WAX (30 m × 0.25 mm I.D., 0.25 μm film thickness) capillary column and fatty acid methyl esters were separated at constant flow [19]. The ion trap mass spectrometer was operated under electron bomb ionisation (EI) mode. Mass spectra of m/z 30–450 were collected using full scan mode with 0.58 s/scan velocity. Solvent delay time was 5 min, source temperature was 230 °C with the electron energy at 70 eV, limit of detection (LOD) was defined as lowest concentrations with signal-to-noise (S/N) ratios of 10 and all calibration samples showed high repeatability (Additional file 1: Tables S1 and S2).

### Diagnostic criteria

NAFLD in this study was defined using the guidelines provided by the Chinese Association for Study of Liver Diseases [20]. Briefly, ultrasonographic evaluation of the liver (considering the size contour, structure, posterior beam attenuation and echogenicity) for fatty infiltration was carried out (using a 3.5 MHz probe Model: SSI – 8000, Phillip, Netherlands) by an ultrasonographist blinded to both respondents’ serum biochemical profile and disease history.

In this study, NAFLD is diagnosed provided histopathological reports of liver biopsies fulfills the pathological diagnostic criteria for fatty liver disease in the absence of; (a) specific disease(s) that could trigger liver steatosis; viral hepatitis (HBV/HCV), hepatolenticular degeneration, autoimmune diseases or/and a history of total parenteral nutrition, or intake of hepatotoxic-inducing drugs (e.g., tamoxifen, amiodarone, sodium valproate, methotrexate, and glucocorticoid), (b) history of habitual alcohol consumption or ethanol intake of <140 g/week or <70 g/week for men and women respectively.

Furthermore, patients who satisfy the above criteria for NAFLD in our study were additional categorized by BMI i.e. lean – NAFLD (BMI: 18.5–24.0 kg/m^2^), overweight – NAFLD (BMI: 24.0–28.0 kg/m^2^) and obese – NAFLD (BMI ≥ 28.0 kg/m^2^).

### Statistical analysis

All data were presented as mean ± standard deviation (SD) and proportion. Continuous and categorical variables were analysed by one-way ANOVA and Chi-square test respectively. Distinction in FFA patterns among HC, lean –, overweight – and obese – NAFLD patients were explored using one-way analysis of covariance (ANCOVA) adjusting for age and sex in addition to Bonferroni post hoc test to investigate distinctions between any two groups. Partial correlation analysis was adopted to explore the relationship between FFA and biochemical indicators, after adjusting for age and sex, in all participants. Receiver operating characteristics (ROC) analysis was used and areas under the ROC curve (AUC) were calculated to compare the effectiveness of different FFA to identify NAFLD. Logistic regression models were adopted to evaluate the odd ratio (OR) and 95% confidential interval (CI) of FFA with higher AUC and Youden’s index (the first three) for NAFLD of higher concentrations than their cut-off values.

SPSS 18.0 (Beijing Stats Data Mining Co. Ltd., Beijing, China) was used for statistical analysis and a *P* value <0.05 (two-tailed) was considered statistically significant.

## Results

### Demographic and biochemical information

Table 1 describes the demographic characteristics and biochemical indicators of the HC and NAFLD patients. While most demographic and biochemical indicators were significantly different among HC and NAFLD patients, age, BP, TC, LDL-C, ALT, AST, ALP and GGT were insignificantly different among the three NAFLD groups. Contrariwise, FBG, TG, TC, blood lipids, ALT, AST and GGT were significantly higher among the three NAFLD groups compared HC.

**Table 1:** Demographic, anthropometric and clinical characteristics of all participants

	HC (*n* = 66)	NAFLD Patients		
		Lean (*n* = 67)	Overweight (*n* = 55)	Obese (*n* = 48)
Sex *(m/f)*	42/24	41/26	36/19	30/18
Age (*y*)	40.03 ± 10.17	48.38 ± 12.13^a^	47.74 ± 13.30^a^	49.18 ± 12.25^a^
BMI (*kg/m* ^*2*^)	20.41 ± 2.06	22.58 ± 0.75^a^	26.19 ± 1.03^ab^	29.49 ± 2.17^abc^
WC (*cm*)	78.23 ± 9.11	88.25 ± 6.03^a^	93.51 ± 7.79^ab^	98.06 ± 9.65^ab^
Body fat (*%*)	20.23 ± 4.82	25.25 ± 4.93^a^	30.79 ± 3.90^ab^	31.32 ± 3.07^ab^
SBP (*mmHg*)	114.74 ± 10.44	131.91 ± 19.92^a^	130.87 ± 14.90^a^	126.44 ± 10.55^a^
DBP (*mmHg*)	79.42 ± 7.93	84.75 ± 7.57^a^	88.30 ± 8.72^a^	85.94 ± 6.53^a^
FBG (*mmol/L*)	5.28 ± 0.46	6.22 ± 1.66^a^	5.54 ± 0.70^b^	6.28 ± 1.33^ac^
Fasting insulin (*IU/ml*)	5.79 ± 2.77	8.16 ± 3.14^a^	8.91 ± 4.35^a^	11.89 ± 5.76^abc^
HOMA-IR	1.38 ± 0.74	2.33 ± 1.15^a^	2.18 ± 1.10^a^	3.59 ± 2.82^abc^
TC (*mmol/L*)	4.40 ± 0.66	5.31 ± 0.93^a^	5.19 ± 0.77^a^	5.35 ± 1.07^a^
TG (*mmol/L*)	0.86 ± 0.30	1.68 ± 0.66^a^	1.87 ± 1.13^a^	2.17 ± 1.27^ab^
HDL-C (*mmol/L*)	1.69 ± 0.31	1.51 ± 0.27^a^	1.54 ± 0.36^a^	1.31 ± 0.26^abc^
LDL-C (*mmol/L*)	2.50 ± 0.52	3.29 ± 0.78^a^	3.20 ± 0.73^a^	3.30 ± 0.86^a^
ALT (*U/L*)	15.21 ± 7.55	24.06 ± 10.92^a^	25.11 ± 15.84^a^	28.11 ± 14.94^a^
AST (*U/L*)	18.65 ± 4.45	22.21 ± 6.10^a^	23.92 ± 7.93^a^	22.15 ± 6.09^a^
ALP (*U/L*)	64.56 ± 15.87	72.88 ± 17.96^a^	67.61 ± 15.54	69.19 ± 19.34
GGT (*U/L*)	19.66 ± 12.93	46.32 ± 40.05^a^	45.84 ± 43.95^a^	48.48 ± 24.75^a^

^a^ *P* < 0.05, when compared with healthy controls;

^b^ *P* < 0.05, when compared with Lean-NAFLD group;

^c^ *P* < 0.05, when compared with overweight-NAFLD group

Additionally, biochemical indicators were insignificantly different between lean and overweight NAFLD subjects, but fasting insulin and HOMA-IR were significantly higher and HDL-c was significantly lower among obese-NAFLD than lean-NAFLD or overweight-NAFLD.

### Plasma FFA levels

The GC-MS method had excellent linearity and repeatability and bias% are within acceptable range. Serum profiles of FFA in HC and NAFLD patients are summarised in Table 2. NAFLD patients expressed significantly higher profiles of 12 FFA and total FFA than HC. There was no significant difference in all FFA and total FFA profiles between lean and overweight NAFLD patients. However, obese NAFLD patients presented significantly higher 14:0, 16:0, 16:1, 18:1 and total FFA profiles than HC. In addition, FFA profiles differed insignificantly after stratifying sex of participants Additional file 1: (Table S3).

**Table 2:** FFA profiles of healthy controls and NAFLD patients^*^

	HC (*n* = 66)	NAFLD Patients		
FFA (μg/ml)		Lean (*n* = 67)	Overweight (*n* = 55)	Obese (*n* = 48)
14:0	5.00 ± 2.49	7.65 ± 3.25^a^	7.13 ± 4.74^a^	11.23 ± 8.14^a b c^
16:0	431.38 ± 102.97	495.62 ± 100.32^a^	478.25 ± 119.66	571.50 ± 190.60^a b c^
16:1	29.52 ± 13.56	42.65 ± 16.93^a^	44.21 ± 28.85^a^	54.49 ± 30.27^a b c^
18:0	165.75 ± 49.83	200.46 ± 57.69^a^	193.95 ± 53.61^a^	206.68 ± 59.56^a^
18:1	240.53 ± 92.15	286.83 ± 65.19^a^	293.28 ± 103.12^a^	346.61 ± 118.96^a b c^
18:2	817.38 ± 266.00	898.36 ± 179.39^a^	880.29 ± 146.17	942.46 ± 202.84^a^
18:3	33.49 ± 12.62	46.54 ± 23.59^a^	49.87 ± 24.93^a^	50.09 ± 26.22^a^
γ-18:3	13.40 ± 7.15	22.20 ± 10.92^a^	20.43 ± 12.66^a^	20.30 ± 11.48^a^
20:4	142.71 ± 39.80	164.67 ± 42.92^a^	162.05 ± 46.79	158.19 ± 68.43
20:5	9.74 ± 8.54	12.43 ± 5.61	16.90 ± 32.62	21.33 ± 31.26^a^
22:5	5.38 ± 2.45	8.38 ± 4.67^a^	9.28 ± 7.17^a^	10.42 ± 3.99^a^
22:6	179.32 ± 61.94	223.62 ± 65.28^a^	222.52 ± 61.41^a^	257.80 ± 133.98^a b^
Total FFA	2093.33 ± 558.11	2426.09 ± 465.28^a^	2420.81 ± 555.18^a^	2739.01 ± 810.35^a b c^

^*^All *P-values* were tested using ANCOVA and Bonferroni post – hoc test adjusting for sex and age

^a^ *P* < 0.05, when compared with healthy controls;

^b^ *P* < 0.05, when compared with lean – NAFLD group;

^c^ *P* < 0.05, when compared with overweight – NAFLD group

### Correlation analysis

Twelve FFA alongside total FFA positively correlated with FBG, blood lipid (TC, TG) and liver enzymes (ALT, GGT) (Table 3, Additional file 1: Table S4). Six, nine and eleven FFA positively correlated with BP (SBP and DBP), fasting insulin and HOMA-IR respectively. Two FFA positively correlated with HDL-C, while none with ALP. While the correlations were distributed between biochemical indicators and FFA among HC only (Additional file 1: Table S5), most FFA were significantly correlated with blood lipid; TC, TG and HDL-C among lean NAFLD patients only (Additional file 1: Table S6). Furthermore, TC, TG, ALT and AST correlated with FFA in overweight NAFLD patients only (Additional file 1: Table S7), and TG significantly correlated with FFA among obese-NAFLD patients (Additional file 1: Table S8).

**Table 3:** Correlation analyses between FFA and metabolic indicators of all participants^*^

	14:0	16:0	16:1	18:0	18:1	18:2	γ-18:3	18:3	20:4	20:5	22:5	22:6	Total FFA
BMI	+	+	+	+	+	NS	+	+	NS	+	+	+	+
WC	+	+	+	+	+	NS	+	+	NS	+	+	NS	+
Body fat	+	+	+	+	+	+	+	+	NS	+	+	+	+
SBP	NS	+	NS	+	NS	+	NS	NS	NS	+	+	+	+
DBP	NS	+	NS	+	NS	+	NS	NS	+	+	NS	+	+
FBG	+	+	+	+	+	+	+	+	+	+	+	+	+
Fasting insulin	+	+	+	+	+	+	+	+	+	NS	NS	NS	+
HOMA-IR	+	+	+	+	+	+	+	+	+	+	NS	+	+
TC	+	+	+	+	+	+	+	+	+	+	+	+	+
TG	+	+	+	+	+	+	+	+	+	+	+	+	+
HDL-C	NS	NS	NS	NS	NS	NS	NS	−	+	NS	NS	NS	NS
LDL-C	+	+	+	+	+	+	+	NS	+	+	+	+	+
ALT	+	+	+	+	+	+	+	+	+	+	+	+	+
AST	+	+	+	+	+	NS	NS	NS	+	NS	+	+	+
ALP	NS	NS	NS	NS	NS	NS	NS	NS	NS	NS	NS	NS	NS
GGT	+	+	+	+	+	+	+	+	+	+	+	+	+

*NS* not significant;

+: positive association;

-: negative association between FFA and indictors;

^*^Correlation coefficients and statistical significance are reported in SM Table S4

### Areas under the ROC curve of 12 FFA

The cut-off value, sensitivity, specificity, Youden’s index and area under the ROC curve (AUC) are presented in Table 4. 14:0, 16:1 and γ-18:3 presented AUC of 0.70; 0.74(0.67–0.82), 0.73(0.66–0.81) and 0.74(0.67–0.81), respectively. AUC of 18:0 and 18:3 were 0.70(0.62–0.79) and 0.70(0.62–0.77) respectively, while that of the remaining FFA ranged from 0.60 to 0.70. Youden’s index of 3 FFA; 14:0, 16:1 and 22:5, were higher than 40.00 (43.81, 41.01 and 40.91 respectively). Also, Youden’s index of 7 FFA; 16:0, 18:0, 18:1, 18:2, γ-18:3, 18:3, 22:6 and total FFA ranged from 30.00 to 40.00, and that of 2 FFA; 20:4 and 20:5 were lower than 30.00 (24.24 and 25.19 respectively).

**Table 4:** Sensitivity, Specificity, Youden’s index and area under the curve (AUC) of FFA to predict NAFLD

FFA	Cut-off value	Sensitivity (%)	Specificity (%)	Youden index	AUC 95% CI
14:0	5.74	69.74	74.07	43.81	0.74(0.67–0.82)
16:0	486.64	55.92	81.48	37.4	0.69(0.60–0.77)
16:1	31.02	74.34	66.67	41.01	0.73(0.66–0.81)
18:0	179	62.5	74.07	36.57	0.70(0.62–0.79)
18:1	274.39	55.92	79.63	35.55	0.69(0.60–0.78)
18:2	774.88	75.66	59.26	34.92	0.64(0.54–0.74)
γ-18:3	12.06	81.58	55.56	37.13	0.74(0.67–0.81)
18:3	36.8	65.13	68.52	33.65	0.70(0.62–0.77)
20:4	161.71	42.76	81.48	24.24	0.63(0.54–0.71)
20:5	11.9	38.16	87.04	25.19	0.64(0.56–0.72)
22:5	9.49	42.76	98.15	40.91	0.66(0.58–0.73)
22:6	161.28	88.82	46.3	35.11	0.69(0.61–0.78)
Total FFA	2167.89	65.13	68.52	33.65	0.70(0.61–0.79)

### Logistic regression analysis

The OR (95% CI) for NAFLD of 4 FFA; 14:0, 16:1, γ-18:3 and 22:5 are summarized in Table 5. After adjusting for confounding variables (age, sex, BMI, smoking, alcohol consumption and regular exercise), OR (95% CI) of 14:0 and 16:1 were 5.58(1.37, 22.76) and 4.36(1.34, 14.13) respectively. In addition, OR of γ-18:3 and 22:5 were statistically insignificant in crude and multivariable adjusted logistic regression models.

**Table 5:** Odds ratio and 95% CI for NAFLD of 14:0, 16:1, γ-18:3 and 22:5

	Model 1	Model 2	Model 3
14:0	2.90(1.20, 6.99)	6.69(2.38, 18.80)	5.58(1.37, 22.76)
16:1	2.95(1.34, 6.51)	1.99(1.01, 4.63)	4.36(1.34, 14.13)
γ-18:3	1.97(0.91, 4.27)	1.10(0.44, 2.72)	0.50(0.14, 1.72)
22:5	2.15(0.97, 5.92)	1.82(0.79, 4.63)	1.03(0.45, 1.83)

Model 1: Crude OR;

Model 2: Adjusted for age, sex and BMI;

Model 3: Adjusted for age, sex, BMI, smoking, alcohol consumption and regular exercise

## Discussion

In the current study, we identified four groups according to subjects’ BMI and NAFLD status (HC, lean – NAFLD, overweight – NAFLD and obese – NAFLD), and firstly analysed the FFA profiles of all NAFLD patients and HC. All NAFLD patients had higher serum concentrations of all types of and total FFA than HC. Lean and overweight NAFLD had similar concentrations in all FFA, but obese NAFLD patients presented significantly higher levels of all types of FFA. All FFA were positively associated with glucose, ALT, GGT, TC and TG, (independent of age and sex). These biochemical indices are likely important in the diagnosis of NAFLD. In addition, we observed 14:0 and 16:1 as unique FFA of prominent diagnostic importance for early screening NAFLD.

NAFLD is the most common spectrum of liver disease in many countries [21]. It is a hepatic manifestation of metabolic syndrome [22] associated with serious metabolic and liver-related complications. Lipid accumulation is one of the important etiologies and pathogenesis of NAFLD and its complications [23]; FFA fluxes from adipose tissue increases, leading to lipotoxicity in the development of NAFLD [24]. Thus, FFA profiles in NAFLD patients are different from those of HC.

In the current study, serum FFA profiles were significantly higher in NAFLD patients and positively associated with almost all metabolic indicators, especially blood glucose, lipid and liver enzymes. The positive association between serum FFA, fasting insulin and HOMA-IR may be explained by the inhibitory effects of FFA on glycogenolysis, thus leading to elevated insulin levels and hepatic IR [25].

On the other hand, persistent elevation of FFA is likely to also increase gluconeogenesis [25] and inhibit the phosphorylation and oxidation of glucose, thus leading to high blood glucose and IR [26]. IR is a critical risk factor for many metabolic diseases [27], thus, elevated FFA could be observed in many metabolic diseases, such as obesity, hyperlipidemia, cardiovascular disease and diabetes which are often positively associated with plasma FFA [25, 28, 29]. Moreover, obesity is an established risk factor for NAFLD [30], and high body fat always increases FFA concentrations [31]. In tandem with our present study, all NAFLD patients had higher FFA, and obese patients had the poorest FFA profile.

Comprised hepatic lipid uptake has been linked to severe lipid accumulation in hepatic cells. In a recent report by Gu et al. [32], hepatic steatosis was observed in solute carrier 7a3a (*slc7a3a)* – null mutants zebra fish hepatocyte and fasted cultured human hepatocytes. *slc7a3a* is one of the many kinds of specialized protein on the plasma membrane vital for intracellular transport with inhibited expression during the fasting. As a result, nitric oxide – mediated *cyclic* guanosine mono phosphate synthesis which acts upstream of adenosine mono phosphate (AMP) – activated protein kinase to regulate lipid oxidation are inhibited thereby leading to anomalous lipid retention in the hepatocytes [32].

Among the 12 FFA evaluated in our study, 14:0 and 16:1 proves more effective than other FFA in predicting NAFLD. 14:0 is a long chain saturated fatty acid, predominantly produced from fatty acid synthase (FAS) pathway. A small proportion of 14:0 is synthesised from the shortening of 16:0 by peroxisomal β-oxidation [33]. It could inhibit endothelial cells growth, induce proinflammatory responses and increase the risk of cardiovascular complications [34]. Moreover, its molecular percentage and concentration of serum cholesteryl-myristate are useful in prediction of serum TC [35]. 14:0 has also been recommended as a marker likely useful to decipher between simple steatosis and non-alcoholic steatohepatitis [36].

16:1 is a product of stearoyl-CoA desaturase (SCD), and could reflect the activity of SCD. Circulating 16:1 is associated with endogenous sources (liver, adipose tissue) of fatty acid synthesis [37]. It is strongly associated with TG and IR and observed to be higher in patients with hypertriglyceridemia, thus, has been reported as an independent marker of triglyceridemia. Elevated serum 16:1 profile is also significantly related to abdominal adiposity and fatty liver disease (FLD), which is a likely reflection of SCD activation [38] and hepatic lipogenesis [39].

Dietary pattern exposure is a vital component as to either improving or compromising vascular health. In a review by Scicchitano et al. [40], the beneficial role of functional traditional foods in the management of dyslipidemia was extensively reported. For example, the beneficial effect of Mediterranean diet in improving cardio-metabolic health and plummeting cardiovascular-related morbidities and mortalities has been reported [41, 42]. Though the precise pathophysiology of how these foods exert beneficial effect is largely unclear, it is likely not far-fetched from down-regulating the action of 7α- hydroxylase (a rate limiting enzyme in bile synthesis and cholesterol regulation), thereby increasing faecal excretion of cholesterol. Limited cholesterol level in vivo, potentially allows 3-hydroxy-3-methyl-glutaryl-coenzyme A reductase activate the expression of LDL receptors to upregulate the breakdown of LDL and reduction in cholesterol levels in mammalian cells [40].

In tandem with our finding, diary fat – a rich source of 14:0 has been reported to acutely raise LDL levels. Zock et al. [43] in a 3-week human trial involving the administration of dietary saturated fatty acids observed acute elevation of LDL and ApoB levels with decreased HDL level by 14:0 and 16:0. That report alongside our findings lends credence to likely deleterious potential of 14:0 and 16:1 strongly associated with NAFLD, which reflects the important role of these two FFA in NAFLD and many metabolic disorders as they possess promising diagnostic value in the management of NAFLD.

There are some limitations worth mentioning in our current study. Both 14:0 and 16:1 can be derived from diets and whether their dietary intakes were associated with serum levels were not studied. Even though FFA profiles stratified by sex were insignificantly different (Additional file 1: Table S3) in our study, other studies [44, 45] have reported otherwise. More males than females participated in this study, but potential adjustment for sex in our final analyses downplayed the likely confounding effect. On the other hand, the study population was recruited from the northern part of China alongside small sample size. Thus our findings may lack generality to the whole population. Multi-ethnic longitudinal studies are necessary to validate our findings.

## Conclusion

In conclusion, serum FFA profiles of NAFLD patients were significantly higher compared with HC, and obese NAFLD patients presented the poorest FFA profiles. Furthermore, 14:0 (myristic acid) and 16:1 (palmitoleic acid) are of promising diagnostic value in the early diagnosis of NAFLD especially among normal weight individuals.

## Additional file

Additional file 1: Supplementary Information. (DOCX 61 kb)

## Abbreviations

ALP: Alkaline phosphatase
ALT: Alanine aminotransferase
AST: Aspartate aminotransferase
AUC: Area under the curve
BMI: Body mass index
BUN: Blood urea nitrogen
CI: Confidence interval
CREA: Serum creatinine
DBP: Diastolic blood pressure
FBG: Fasting blood glucose
FFA: Free fatty acid
FLD: Fatty liver disease
GC-MS: Gas chromatography-mass spectrometry
GGT: Gamma-glutamyl transpeptidase
HDL-C: High-density lipoprotein cholesterol
HOMA-IR: Homeostasis model assessment insulin resistance
LCPUFA: Long-chain *n*-3 polyunsaturated fatty acids
LDL-C: Low-density lipoprotein cholesterol
LOD: Limit of detection
NAFLD: Non-alcoholic fatty liver disease
OR: Odd ratios
ROC: Receiver operating characteristics
SBP: Systolic blood pressure
TC: Total cholesterol
TG: Triglyceride
UA: Serum uric acid
WC: Waist circumference
