# Supplementary material for: Free fatty acids profile among lean, overweight and obese non-alcoholic fatty liver disease patients: a case – control study
Source: Lipids Health Dis. 2017 Sep 4;16:165. doi: 10.1186/s12944-017-0551-1 (PMC5584533; doi:10.1186/s12944-017-0551-1)
Supplement: Additional file 1: — Supplementary Information. (DOCX 61 kb) [file 12944_2017_551_MOESM1_ESM.docx]

**Additional File**

Rennan Feng^a 1 *^, Chao Luo^b 1^, Chunlong Li^c 1^, Shanshan Du ^a 1^, Akinkunmi Paul Okekunle^a^，Yanchuan Li^a^，Yang Chen^a^, Tianqi Zi^a^, Yucun Niu^a^

**Free Fatty Acids Profile among Lean, Overweight and Obese Non-Alcoholic Fatty Liver Disease patients: A case – control study**

**Institutional Affiliations**

^a^ Department of Nutrition and Food Hygiene, School of Public Health, Harbin Medical University, Harbin 150081, Heilongjiang Province, China

^b^ STD & AIDS Center, Harbin Center for Disease Control and Prevention, Harbin 150056, Heilongjiang Province, China

^c^ Department of General Surgery, the Second Affiliated Hospital of Harbin Medical University, Harbin 150081, Heilongjiang Province, China

^1^ Authors contributed equally to this work.

* **Corresponding author:**

Dr. Rennan Feng

Department of Nutrition and Food Hygiene, School of Public Health, Harbin Medical University; 157 Baojian Street, Nangang District, Harbin 150081, Heilongjiang Province, China. Telephone: +86 0451 87502801; Fax: +86 0451 87502724;

E-mail: fengrennan@163.com;

**Keywords:** FFAs; Body weight; NAFLD; GC-MS, Myristic acid, Palmitoleic acid

**Figure S1: Flowchart describing the recruitment strategy of respondents in the study**

**Lean – NAFLD**

(n = 67)

(18.5 kg/m^2^ ≤ BMI < 24.0 kg/m^2^)

**Overweight – NAFLD**

(n = 55)

(24.0 kg/m^2^ ≤ BMI < 28.0 kg/m^2^)

**Overweight – NAFLD**

(n = 48)

(BMI ≥ 28.0 kg/m^2^)

**Healthy Controls (HC)**

(n = 66)

(18.5 kg/m^2^ ≤ BMI < 24.0 kg/m^2^)

**Serum Free Fatty Acids Assessment using GC – MS**

**Estimation of distinction in biochemical indices and Free Fatty Acid Profiles among the 4 groups**

**NAFLD Patients (n = 170)**

**236** Respondents

Newly diagnosed cases of NAFLD through ultrasonic evaluation of liver by an ultrasonographist blinded to both respondents’ serum biochemical profile and history

**320** participants were recruited at Physical Examinations Center of the Second Affiliated Hospital, Harbin Medical University from March 12, 2013 to April 20, 2013.

84 subjects were excluded as a result of:

- History of alcohol consumption and malignancies
- Pregnancy
- Long-term use of estrogen, taximofen or corticosteroids
- Co-morbidities of other disease(s) aside NAFLD.

| Table S1: The equation and the correlation coefficient of fatty acids | | | | |
| --- | --- | --- | --- | --- |
| FFA | Calibration equation | Regression coefficient | Range of quality (μg/ml) | Limit of detection (μg/ml) |
| 14:0 | Y=0.4414X-0.2206 | 0.9964 | 2.96-25.00 | 0.05 |
| 16:0 | Y=0.0289X+0.481 | 0.9957 | 249.78-750.5 | 0.03 |
| 16:1 | Y=0.0425X-0.0822 | 0.9996 | 25.10-100.10 | 0.08 |
| 18:0 | Y=0.0248X-0.0479 | 0.9992 | 61.00-253.00 | 0.03 |
| 18:1 | Y=0.0319X-0.4984 | 0.9991 | 150.00-750.00 | 0.04 |
| 18:2 | Y=0.0331X-0.2653 | 0.9999 | 250.00-1000.00 | 0.05 |
| γ-18:3 | Y=0.2107X-0.4323 | 0.9995 | 5.30-25.60 | 0.08 |
| 18:3 | Y=0.0689X-0.0033 | 0.9981 | 1.10-50.00 | 0.08 |
| 20:4 | Y=0.0269X-0.034 | 0.9997 | 50.00-300.00 | 0.05 |
| 20:5 | Y=0.0242X+0.0328 | 0.9953 | 4.90-50.00 | 0.05 |
| 22:5 | Y=0.0069X+0.0051 | 0.9975 | 2.50-56.00 | 0.06 |
| 22:6 | Y=0.0241X-0.1348 | 0.9993 | 20.00-100.00 | 0.07 |
| ^*^ X: the quality of fatty acids; Y: relative peak area = peak area of fatty acid/peak area of internal standard. | | | | |

| Table S2: The repeatability of the calibration samples (n=6) | | | | |
| --- | --- | --- | --- | --- |
| FFA | True concentration | Measured concentration | CV% | Bias% |
| 14:0 | 5.00 | 4.64±0.24 | 5.17 | 92.80 |
| 16:0 | 350.00 | 352.24±19.26 | 5.47 | 100.61 |
| 16:1 | 50.00 | 48.17±1.69 | 3.51 | 96.34 |
| 18:0 | 150.00 | 142.38±8.29 | 5.82 | 94.90 |
| 18:1 | 300.00 | 304.26±17.36 | 5.71 | 101.42 |
| 18:2 | 450.00 | 443.37±34.28 | 7.73 | 98.53 |
| γ-18:3 | 15.00 | 14.02±0.91 | 6.49 | 93.47 |
| 18:3 | 10.00 | 9.34±0.56 | 6.00 | 93.40 |
| 20:4 | 150.00 | 147.67±8.67 | 5.87 | 98.45 |
| 20:5 | 5.00 | 5.19±0.37 | 7.13 | 103.80 |
| 22:5 | 10.00 | 9.07±0.26 | 2.87 | 90.70 |
| 22:6 | 50.00 | 48.05±3.07 | 6.39 | 96.10 |
| Values were shown as mean±SD; FFA concentration: μg/ml | | | | |

| Table S3: Lean/Overweight/Obese – NAFLD and serum FFA profiles stratified by sex | | | | |
| --- | --- | --- | --- | --- |
|  |  | Male (149) | Female (87) | *P* value |
| Age (y) | | 45.52±12.4 | 47.72±12.55 | 0.24 |
| **NAFLD patients** | |  |  |  |
|  | Lean | 41 | 26 | 0.97 |
|  | Overweight | 36 | 19 |  |
|  | Obese | 30 | 18 |  |
| **Serum FFA profile** | | |  |  |
|  | 14:0 | 7.93±5.86 | 6.36±3.04 | 0.07 |
|  | 16:0 | 494.82±150.08 | 475.03±95.17 | 0.38 |
|  | 16:1 | 44.53±25.86 | 33.86±16.36 | 0.01 |
|  | 18:0 | 188.22±59.24 | 195.44±50.38 | 0.44 |
|  | 18:1 | 293.51±109.24 | 270.31±73.03 | 0.16 |
|  | 18:2 | 869.35±212.88 | 908.22±203.11 | 0.26 |
|  | 18:3 | 45.63±25.21 | 40.43±14.51 | 0.16 |
|  | γ-18:3 | 18.61±10.95 | 19.43±11.34 | 0.65 |
|  | 20:4 | 160.21±54.37 | 145.80±33.10 | 0.08 |
|  | 20:5 | 15.65±24.92 | 11.41±9.96 | 0.24 |
|  | 22:5 | 8.27±5.45 | 6.94±3.37 | 0.12 |
|  | 22:6 | 222.19±93.80 | 205.15±63.67 | 0.23 |
|  | Total FFA | 2373.61±675.71 | 2351.07±449.31 | 0.83 |

| Table S4: Partial correlation^†^ analyses between FFAs and biochemical indicators of all participants | | | | | | | | | | | | | |
| --- | --- | --- | --- | --- | --- | --- | --- | --- | --- | --- | --- | --- | --- |
|  | 14:0 | 16:0 | 16:1 | 18:0 | 18:1 | 18:2 | γ-18:3 | 18:3 | 20:4 | 20:5 | 22:5 | 22:6 | Total FFAs |
| BMI | 0.291^**^ | 0.211^**^ | 0.245^**^ | 0.165^*^ | 0.273^**^ | 0.137 | 0.145^*^ | 0.237^**^ | 0.023 | 0.266^**^ | 0.312^**^ | 0.186^**^ | 0.227^**^ |
| WC | 0.434^**^ | 0.288^**^ | 0.412^**^ | 0.261^**^ | 0.332^**^ | 0.156 | 0.270^**^ | 0.325^**^ | 0.072 | 0.164^*^ | 0.238^**^ | 0.162 | 0.288^**^ |
| Body fat | 0.298^**^ | 0.308^**^ | 0.239^**^ | 0.290^**^ | 0.284^**^ | 0.285^**^ | 0.239^**^ | 0.261^**^ | 0.097 | 0.226^**^ | 0.341^**^ | 0.275^**^ | 0.385^**^ |
| SBP | 0.136 | 0.195^*^ | 0.101 | 0.194^*^ | 0.115 | 0.200^*^ | 0.095 | 0.128 | 0.140 | 0.208^*^ | 0.235^**^ | 0.308^**^ | 0.255^**^ |
| DBP | 0.152 | 0.199^*^ | 0.128 | 0.211^*^ | 0.127 | 0.171^*^ | 0.122 | 0.150 | 0.177^*^ | 0.189^*^ | 0.152 | 0.271^**^ | 0.215^*^ |
| FBG | 0.225^**^ | 0.321^**^ | 0.220^**^ | 0.246^**^ | 0.328^**^ | 0.281^**^ | 0.170^*^ | 0.289^**^ | 0.271^**^ | 0.299^**^ | 0.245^**^ | 0.328^**^ | 0.371^**^ |
| Fasting insulin | 0.382^**^ | 0.219^**^ | 0.194^**^ | 0.229^**^ | 0.277^**^ | 0.218^**^ | 0.131^*^ | 0.366^**^ | -0.046 | 0.050 | 0.062 | 0.058 | 0.251^**^ |
| HOMA-IR | 0.404^**^ | 0.273^**^ | 0.256^**^ | 0.247^**^ | 0.346^**^ | 0.247^**^ | 0.147^*^ | 0.440^**^ | 0.036 | 0.134^*^ | 0.109 | 0.159^*^ | 0.317^**^ |
| TC | 0.362^**^ | 0.375^**^ | 0.428^**^ | 0.490^**^ | 0.332^**^ | 0.365^**^ | 0.362^**^ | 0.211^**^ | 0.477^**^ | 0.274^**^ | 0.383^**^ | 0.337^**^ | 0.463^**^ |
| TG | 0.611^**^ | 0.600^**^ | 0.652^**^ | 0.550^**^ | 0.658^**^ | 0.397^**^ | 0.398^**^ | 0.517^**^ | 0.316^**^ | 0.287^**^ | 0.488^**^ | 0.356^**^ | 0.565^**^ |
| HDL-C | -0.123 | -0.006 | -0.040 | 0.088 | -0.081 | 0.034 | -0.023 | -0.154^*^ | 0.146^*^ | -0.020 | -0.036 | 0.054 | -0.005 |
| LDL-C | 0.209^**^ | 0.186^**^ | 0.209^**^ | 0.284^**^ | 0.140^*^ | 0.233^**^ | 0.223^**^ | 0.100 | 0.293^**^ | 0.177^**^ | 0.207^**^ | 0.172^**^ | 0.288^**^ |
| ALT | 0.304^**^ | 0.295^**^ | 0.302^**^ | 0.293^**^ | 0.321^**^ | 0.163^*^ | 0.170^**^ | 0.235^**^ | 0.292^**^ | 0.203^**^ | 0.259^**^ | 0.265^**^ | 0.346^**^ |
| AST | 0.156^*^ | 0.158^*^ | 0.164^*^ | 0.214^**^ | 0.163^*^ | 0.080 | 0.114 | 0.032 | 0.252^**^ | 0.099 | 0.163^*^ | 0.173^**^ | 0.190^**^ |
| ALP | 0.039 | 0.027 | 0.052 | 0.047 | -0.009 | 0.037 | -0.003 | 0.003 | -0.051 | 0.005 | -0.020 | -0.046 | 0.001 |
| GGT | 0.374^**^ | 0.363^**^ | 0.473^**^ | 0.381^**^ | 0.389^**^ | 0.183^**^ | 0.235^**^ | 0.263^**^ | 0.338^**^ | 0.430^**^ | 0.337^**^ | 0.360^**^ | 0.370^**^ |
| ^†^ - partial correlation coefficients adjusted for age and sex  ^*^ *P*<0.05, ^**^ *P*<0.01 | | | | | | | | | | | | | |

| Table S5: Partial correlation^†^ analyses between FFAs and biochemical indicators among healthy controls only | | | | | | | | | | | | | |
| --- | --- | --- | --- | --- | --- | --- | --- | --- | --- | --- | --- | --- | --- |
|  | 14:0 | 16:0 | 16:1 | 18:0 | 18:1 | 18:2 | γ-18:3 | 18:3 | 20:4 | 20:5 | 22:5 | 22:6 | Total FFAs |
| BMI | -0.421^**^ | -0.388^**^ | -0.398^**^ | -0.564^**^ | -0.171 | -0.443^**^ | -0.299^*^ | -0.271^*^ | -0.274^*^ | -0.165 | -0.227 | -0.179 | -0.351^**^ |
| WC | -0.084 | -0.155 | -0.033 | -0.323 | 0.080 | -0.222 | -0.321 | -0.127 | -0.046 | -0.166 | 0.167 | -0.055 | -0.144 |
| Body fat | 0.098 | 0.231 | 0.189 | 0.058 | 0.375^**^ | 0.180 | -0.106 | 0.106 | 0.053 | 0.003 | -0.279^*^ | 0.046 | 0.301^*^ |
| SBP | 0.269 | 0.236 | 0.409 | 0.484^*^ | -0.032 | 0.131 | 0.082 | 0.342 | 0.318 | 0.101 | 0.264 | 0.532^*^ | 0.201 |
| DBP | 0.316 | 0.389 | 0.456^*^ | 0.387 | 0.022 | 0.163 | -0.073 | 0.396 | 0.390 | -0.011 | 0.364 | 0.528^*^ | 0.218 |
| FBG | 0.236 | 0.322^*^ | 0.110 | 0.282^*^ | 0.256 | 0.400^**^ | 0.211 | 0.470^**^ | 0.426^**^ | 0.356^**^ | 0.153 | 0.514^**^ | 0.407^**^ |
| Fasting insulin | -0.004 | 0.013 | 0.069 | 0.139 | 0.346^**^ | 0.286^*^ | -0.195 | 0.483^**^ | 0.038 | 0.097 | -0.042 | 0.040 | 0.169 |
| HOMA-IR | 0.094 | 0.121 | 0.095 | 0.220 | 0.414^**^ | 0.383^**^ | -0.096 | 0.588^**^ | 0.147 | 0.176 | 0.010 | 0.156 | 0.282^*^ |
| TC | 0.229 | 0.187 | 0.296^*^ | 0.279^*^ | 0.083 | 0.120 | 0.196 | 0.049 | 0.267^*^ | 0.243 | 0.330^*^ | 0.163 | 0.215 |
| TG | 0.161 | -0.020 | 0.362^**^ | 0.035 | 0.191 | -0.097 | -0.124 | 0.084 | 0.024 | 0.259 | 0.162 | -0.104 | -0.053 |
| HDL-C | 0.451^**^ | 0.448^**^ | 0.454^**^ | 0.625^**^ | 0.259 | 0.254 | 0.334^*^ | 0.123 | 0.491^**^ | 0.286^*^ | 0.397^**^ | 0.489^**^ | 0.387^**^ |
| LDL-C | -0.023 | -0.074 | 0.060 | -0.062 | -0.053 | -0.062 | 0.141 | -0.070 | -0.079 | 0.068 | 0.237 | -0.161 | -0.022 |
| ALT | 0.280^*^ | 0.286^*^ | 0.049 | .302^*^ | 0.451^**^ | 0.272^*^ | 0.215 | 0.359^**^ | 0.493^**^ | -0.054 | 0.205 | 0.183 | 0.367^**^ |
| AST | 0.249 | 0.209 | 0.097 | 0.133 | 0.311^*^ | 0.018 | 0.252 | 0.141 | 0.361^**^ | -0.087 | 0.104 | 0.267^*^ | 0.219 |
| ALP | -0.206 | -0.190 | -0.167 | -0.172 | -0.303^*^ | -0.233 | -0.078 | -0.162 | -0.078 | 0.092 | 0.154 | 0.019 | -0.245 |
| GGT | 0.376^**^ | 0.402^**^ | 0.257 | 0.237 | 0.300^*^ | 0.220 | -0.037 | 0.260 | 0.539^**^ | 0.349^**^ | 0.195 | 0.118 | 0.316^*^ |
| ^†^ - partial correlation coefficients adjusted for age and sex  ^*^ *P*<0.05, ^**^ *P*<0.01 | | | | | | | | | | | | | |

| Table S6: Partial correlation^†^ analyses between FFAs and biochemical indicators among lean-NAFLD participants only | | | | | | | | | | | | | |
| --- | --- | --- | --- | --- | --- | --- | --- | --- | --- | --- | --- | --- | --- |
|  | 14:0 | 16:0 | 16:1 | 18:0 | 18:1 | 18:2 | γ-18:3 | 18:3 | 20:4 | 20:5 | 22:5 | 22:6 | Total FFAs |
| BMI | 0.297^*^ | 0.090 | 0.088 | 0.050 | 0.033 | -0.020 | -0.097 | 0.234 | -0.259 | 0.072 | -0.166 | -0.078 | 0.038 |
| WC | 0.336 | 0.306 | 0.133 | 0.252 | 0.253 | 0.426^*^ | 0.246 | 0.480^**^ | 0.428^*^ | 0.052 | 0.223 | 0.261 | 0.291 |
| Body fat | 0.298^*^ | 0.345^*^ | 0.264 | 0.310^*^ | 0.221 | 0.288^*^ | 0.304^*^ | 0.084 | 0.089 | 0.181 | 0.337^*^ | 0.242 | 0.300^*^ |
| SBP | 0.413^*^ | 0.374 | 0.455^*^ | 0.104 | 0.367 | 0.336 | 0.024 | 0.154 | 0.049 | 0.020 | 0.345 | 0.218 | 0.311 |
| DBP | 0.374 | 0.425^*^ | 0.303 | -0.024 | 0.263 | 0.377 | 0.141 | 0.084 | 0.117 | -0.043 | 0.422^*^ | 0.222 | 0.316 |
| FBG | 0.150 | 0.148 | 0.170 | -0.010 | 0.286^*^ | -0.080 | -0.073 | 0.193 | -0.042 | -0.064 | -0.060 | -0.184 | 0.046 |
| Fasting insulin | 0.272^*^ | 0.043 | -0.037 | 0.078 | -0.009 | -0.158 | 0.094 | 0.323^*^ | -0.212 | -0.206 | -0.280^*^ | -0.296^*^ | -0.046 |
| HOMA-IR | 0.251 | 0.122 | 0.010 | 0.015 | 0.113 | -0.168 | -0.003 | 0.350^*^ | -0.118 | -0.187 | -0.170 | -0.271 | -0.012 |
| TC | 0.223 | 0.420^**^ | 0.393^**^ | 0.532^**^ | 0.268 | 0.442^**^ | 0.091 | 0.097 | 0.554^**^ | 0.368^**^ | 0.422^**^ | 0.479^**^ | 0.517^**^ |
| TG | 0.564^**^ | 0.667^**^ | 0.612^**^ | 0.541^**^ | 0.686^**^ | 0.213 | 0.264 | .470^**^ | 0.284^*^ | 0.080 | 0.276 | 0.199 | 0.565^**^ |
| HDL-C | 0.143 | 0.353^*^ | 0.447^**^ | 0.383^**^ | 0.350^*^ | 0.401^**^ | 0.237 | -0.186 | 0.326^*^ | 0.212 | 0.272 | 0.278^*^ | 0.389^**^ |
| LDL-C | 0.112 | 0.288^*^ | 0.204 | 0.450^**^ | 0.067 | 0.388^**^ | -0.014 | 0.063 | 0.450^**^ | 0.339^*^ | 0.333^*^ | 0.450^**^ | 0.394^**^ |
| ALT | -0.318^*^ | -0.224 | -0.181 | -0.052 | -0.262 | -0.276^*^ | -0.327^*^ | -0.456^**^ | 0.056 | 0.318^*^ | -0.242 | 0.085 | -0.210 |
| AST | -0.278^*^ | -0.150 | -0.132 | 0.027 | -0.236 | -0.048 | -0.23 | -0.325^*^ | 0.152 | 0.220 | -0.148 | 0.061 | -0.115 |
| ALP | 0.140 | 0.231 | 0.086 | 0.142 | 0.231 | 0.310^*^ | 0.260 | 0.199 | 0.057 | -0.134 | 0.150 | -0.016 | 0.230 |
| GGT | -0.134 | -0.030 | -0.080 | 0.050 | -0.155 | -0.257 | -0.399^**^ | -0.282^*^ | -0.032 | 0.176 | -0.253 | 0.165 | -0.053 |
| ^†^ - partial correlation coefficients adjusted for age and sex  ^*^ *P*<0.05, ^**^ *P*<0.01 | | | | | | | | | | | | | |

| Table S7: Partial correlation^†^ analyses between FFAs and biochemical indicators among overweight-NAFLD participants only | | | | | | | | | | | | | |
| --- | --- | --- | --- | --- | --- | --- | --- | --- | --- | --- | --- | --- | --- |
|  | 14:0 | 16:0 | 16:1 | 18:0 | 18:1 | 18:2 | γ-18:3 | 18:3 | 20:4 | 20:5 | 22:5 | 22:6 | Total FFAs |
| BMI | 0.274 | 0.485^**^ | 0.151 | 0.259 | 0.281 | 0.301^*^ | 0.284 | 0.246 | 0.086 | 0.290 | 0.697^**^ | 0.132 | 0.537^**^ |
| WC | 0.470^**^ | 0.253 | 0.420^*^ | 0.206 | 0.398^*^ | 0.339 | 0.138 | 0.470^**^ | -0.094 | 0.193 | 0.324 | -0.101 | 0.194 |
| Body fat | -0.210 | 0.135 | -0.272 | 0.101 | -0.192 | 0.166 | -0.058 | -0.122 | -0.137 | -0.015 | 0.124 | 0.053 | 0.219 |
| SBP | -0.191 | -0.022 | -0.228 | -0.039 | -0.262 | 0.165 | 0.148 | -0.029 | -0.062 | 0.190 | 0.162 | 0.007 | 0.103 |
| DBP | -0.169 | -0.195 | -0.198 | -0.120 | -0.323^*^ | -0.037 | 0.149 | -0.079 | -0.045 | 0.112 | -0.054 | -0.131 | -0.104 |
| FBG | 0.207 | 0.491^**^ | 0.217 | 0.340^*^ | 0.322^*^ | 0.419^**^ | 0.365^*^ | 0.231 | 0.530^**^ | 0.321^*^ | 0.514^**^ | 0.449^**^ | 0.631^**^ |
| Fasting insulin | 0.400^**^ | 0.131 | 0.157 | 0.410^**^ | 0.147 | 0.230 | 0.219 | 0.105 | 0.111 | 0.060 | 0.128 | 0.184 | 0.331 |
| HOMA-IR | 0.458^**^ | 0.250 | 0.280 | 0.463^**^ | 0.279 | 0.308^*^ | 0.302 | 0.176 | 0.313^*^ | 0.164 | 0.238 | 0.331^*^ | 0.501^**^ |
| TC | 0.429^**^ | 0.516^**^ | 0.455^**^ | 0.623^**^ | 0.487^**^ | 0.524^**^ | 0.653^**^ | 0.279 | 0.624^**^ | 0.267 | 0.590^**^ | 0.325^*^ | 0.748^**^ |
| TG | 0.446^**^ | 0.693^**^ | 0.696^**^ | 0.552^**^ | 0.728^**^ | 0.437^**^ | 0.377^*^ | 0.393^*^ | 0.445^**^ | 0.274 | 0.763^**^ | 0.614^**^ | 0.780^**^ |
| HDL-C | 0.067 | 0.078 | 0.047 | 0.038 | -0.074 | 0.226 | 0.171 | 0.305^*^ | 0.066 | 0.396^**^ | 0.214 | 0.112 | 0.088 |
| LDL-C | 0.136 | 0.135 | -0.032 | 0.287 | 0.088 | 0.275 | 0.298 | -0.023 | 0.247 | -0.084 | 0.140 | -0.123 | 0.354 |
| ALT | 0.527^**^ | 0.721^**^ | 0.698^**^ | 0.580^**^ | 0.738^**^ | 0.469^**^ | 0.457^**^ | 0.537^**^ | 0.525^**^ | 0.150 | 0.681^**^ | 0.337^*^ | 0.723^**^ |
| AST | 0.460^**^ | 0.493^**^ | 0.532^**^ | 0.551^**^ | 0.521^**^ | 0.320^*^ | 0.423^**^ | 0.296 | .390^*^ | 0.207 | 0.510^**^ | 0.260 | 0.571^**^ |
| ALP | 0.050 | 0.155 | 0.065 | 0.206 | 0.083 | 0.240 | -0.035 | 0.116 | -0.059 | 0.090 | 0.164 | -0.072 | 0.124 |
| GGT | 0.484^**^ | 0.459^**^ | 0.722^**^ | .383^*^ | 0.615^**^ | 0.279 | 0.351^*^ | 0.481^**^ | 0.353^*^ | 0.262 | 0.484^**^ | 0.294 | 0.472^**^ |
| ^†^ - partial correlation coefficients adjusted for age and sex  ^*^ *P*<0.05, ^**^ *P*<0.01 | | | | | | | | | | | | | |

| Table S8: Partial correlation^†^ analyses between FFAs and biochemical indicators among obese-NAFLD participants only | | | | | | | | | | | | | |
| --- | --- | --- | --- | --- | --- | --- | --- | --- | --- | --- | --- | --- | --- |
|  | 14:0 | 16:0 | 16:1 | 18:0 | 18:1 | 18:2 | γ-18:3 | 18:3 | 20:4 | 20:5 | 22:5 | 22:6 | Total FFAs |
| BMI | 0.172 | 0.087 | 0.212 | 0.093 | 0.241 | -0.013 | 0.188 | -0.018 | 0.277 | 0.199 | 0.184 | 0.110 | 0.308 |
| WC | 0.811^*^ | 0.991^**^ | 0.991^**^ | 0.919^**^ | 0.955^**^ | 0.883^**^ | 0.000 | 0.937^**^ | -0.234 | 0.054 | 0.432 | 0.847^*^ | 0.901^**^ |
| Body fat | 0.068 | 0.088 | -0.055 | 0.064 | 0.017 | 0.186 | 0.111 | 0.294 | 0.030 | 0.078 | 0.081 | 0.204 | 0.318 |
| SBP | 0.009 | 0.101 | -0.216 | -0.030 | 0.241 | 0.152 | 0.391 | 0.016 | 0.172 | 0.214 | 0.410 | 0.002 | -0.103 |
| DBP | 0.440 | 0.576^*^ | 0.318 | 0.461 | 0.756^**^ | 0.502 | 0.525 | 0.447 | 0.518 | 0.104 | -0.447 | 0.329 | 0.671 |
| FBG | 0.168 | 0.120 | 0.160 | 0.113 | 0.281 | 0.155 | 0.044 | 0.142 | 0.002 | -0.023 | 0.249 | 0.196 | 0.371 |
| Fasting insulin | 0.266 | 0.224 | 0.168 | 0.175 | 0.304 | 0.177 | 0.244 | 0.091 | 0.058 | -0.340^*^ | 0.192 | 0.117 | 0.434^*^ |
| HOMA-IR | 0.233 | 0.185 | 0.158 | 0.135 | 0.311 | 0.119 | 0.169 | 0.189 | 0.025 | -0.267 | 0.237 | 0.171 | 0.425^*^ |
| TC | 0.170 | 0.230 | 0.172 | 0.333 | 0.115 | 0.283 | 0.350^*^ | 0.125 | 0.495^**^ | 0.185 | -0.097 | 0.301 | 0.123 |
| TG | 0.789^**^ | 0.865^**^ | 0.786^**^ | 0.794^**^ | 0.857^**^ | 0.744^**^ | 0.634^**^ | 0.602^**^ | 0.425^*^ | 0.017 | 0.650^**^ | 0.615^**^ | 0.878^**^ |
| HDL-C | -0.192 | -0.153 | -0.154 | -0.006 | -0.277 | -0.201 | -0.103 | -0.351^*^ | 0.269 | -0.049 | -0.576^**^ | -0.110 | -0.493^*^ |
| LDL-C | -0.088 | -0.068 | -0.195 | 0.000 | -0.211 | 0.052 | 0.091 | 0.013 | 0.242 | 0.305 | -0.357 | 0.065 | -0.223 |
| ALT | 0.096 | -0.007 | -0.013 | -0.184 | 0.030 | -0.134 | -0.243 | 0.173 | -0.297 | -0.303 | -0.244 | 0.162 | 0.021 |
| AST | -0.118 | -0.258 | -0.115 | -0.234 | -0.239 | -0.297 | -0.301 | -0.264 | -0.166 | -0.360^*^ | -0.580^**^ | -0.362^*^ | -0.493^*^ |
| ALP | 0.045 | -0.038 | 0.093 | 0.046 | 0.012 | 0.072 | -0.150 | 0.069 | -0.100 | -0.088 | -0.077 | -0.027 | -0.010 |
| GGT | 0.232 | 0.265 | 0.432^**^ | 0.295 | 0.405^*^ | 0.229 | 0.186 | 0.340^*^ | 0.182 | 0.133 | 0.496^*^ | 0.349^*^ | 0.236 |
| ^†^ - partial correlation coefficients adjusted for age and sex  ^*^ *P*<0.05, ^**^ *P*<0.01 | | | | | | | | | | | | | |
